# β-Hydroxybutyric acid improves cognitive function in a model of heat stress by promoting adult hippocampal neurogenesis

**DOI:** 10.1007/s44154-022-00079-6

**Published:** 2022-12-29

**Authors:** Jian Huang, Yongji Wu, Xuejun Chai, Shuai Wang, Yongkang Zhao, Yan Hou, Yue Ma, Shulin Chen, Shanting Zhao, Xiaoyan Zhu

**Affiliations:** 1 College of Veterinary Medicine, Northwest A&F University, Yangling, Shaanxi 712100 People’s Republic of China; 2 Department of Basic Medicine, Xi’an Medical University, Xi’an, Shaanxi 710021 People’s Republic of China

**Keywords:** β-Hydroxybutyric acid, Heat stress, Adult hippocampal neurogenesis, Synaptic plasticity, Cognitive dysfunctions

## Abstract

Heat stress has multiple potential effects on the brain, such as neuroinflammation, neurogenesis defects, and cognitive impairment. β-hydroxybutyric acid (BHBA) has been demonstrated to play neuroprotective roles in various models of neurological diseases. In the present study, we investigated the efficacy of BHBA in alleviating heat stress-induced impairments of adult hippocampal neurogenesis and cognitive function, as well as the underlying mechanisms. Mice were exposed to 43 ℃ for 15 min for 14 days after administration with saline, BHBA, or minocycline. Here, we showed for the first time that BHBA normalized memory ability in the heat stress-treated mice and attenuated heat stress-impaired hippocampal neurogenesis. Consistently, BHBA noticeably improved the synaptic plasticity in the heat stress-treated hippocampal neurons by inhibiting the decrease of synapse-associated proteins and the density of dendritic spines. Moreover, BHBA inhibited the expression of cleaved caspase-3 by suppressing endoplasmic reticulum (ER) stress, and increased the expression of brain-derived neurotrophic factor (BDNF) in the heat stress-treated hippocampus by activating the protein kinase B (Akt)/cAMP response element binding protein (CREB) and methyl-CpG binding protein 2 (MeCP2) pathways. These findings indicate that BHBA is a potential agent for improving cognitive functions in heat stress-treated mice. The action may be mediated by ER stress, and Akt-CREB-BDNF and MeCP2 pathways to improve adult hippocampal neurogenesis and synaptic plasticity.

## Introduction

Adult hippocampal neurogenesis continuously occurs in the subgranular zone (SGZ) of the dentate gyrus of mammals, which comprises several processes, including neural stem cells (NSCs) proliferation, migration, differentiation, survival, and integration of those newly born neurons in the existing neuronal circuits (Altman and Das 1965; Ming and Song 2005). NSCs in the SGZ leave quiescence and sequentially undergo self-renewal and differentiate into neuroblasts, and finally give rise to mature granule neurons (Bonaguidi et al. 2011). The newly generated mature granule neurons integrate into the neural circuitry and endow dentate gyrus with circuits high plasticity. Adult neurogenesis plays a critical role in learning and memory. For instance, ablation of neurogenesis impairs the spatial pattern separation, while enhancement of neurogenesis improves it (Clelland et al. 2009; Creer et al. 2010; Tronel et al. 2012). However, the processes of adult neurogenesis are modulated by numerous local, systemic, and environmental factors, such as neurotransmitters, interneurons, hormones, inflammatory mediators, neurotrophins, and stress (Tozuka et al. 2005; Garza et al. 2012; Kohman and Rhodes 2013; Zhang et al. 2021).

High ambient temperature can lead to heat stress, causing various physiological and pathophysiological responses, and ultimately results in metabolic disorders and neurodegenerative disorders in humans (Kendler et al. 2001; Bongioanni et al. 2021). Meanwhile, heat stress can exacerbate brain dysfunction induced by stroke, traumatic brain injury, and drug consumption (Brown and Kiyatkin 2004; White et al. 2007). Previous studies have illustrated that the brain is extremely susceptive to high temperatures, leading to neuronal death, cognitive dysfunction, neuroinflammation, and memory deficits in rodents (Lee et al. 2015; Chauhan et al. 2021). Studies in animal models of heat stress have also revealed additional changes in the brain, including adult hippocampal neurogenesis (Lee et al. 2015). However, the effects of heat stress on the proliferation of NSCs, as well as the differentiation and migration of newly generated neurons, remains unclear. Hence, additional effort is necessary to link heat stress to adult hippocampal neurogenesis. Moreover, heat stress has adverse impacts on the expressions of synaptic protein in the hippocampus (Erfani et al. 2019). The deficits of adult hippocampal neurogenesis and synaptic plasticity are associated with learning and memory ability (Clelland et al. 2009; Arroyo-García et al. 2021). Indeed, heat stress has been proved to impair learning and memory ability in rodents (Lee et al. 2015; Chauhan et al. 2021). In addition, high-temperature exposure has been demonstrated to exert an inhibitory role in neurogenesis and the development of dendrite (Liu et al. 2012; Wu et al. 2016; Chen et al. 2018; Hood et al. 2018).

The ketone body β-hydroxybutyric acid (BHBA) is not only a simple carrier of energy from the liver to peripheral tissues during prolonged fasting or exercise but also an endogenous histone deacetylase inhibitor, possessing a series of regulatory functions, like gene expression, lipid metabolism, neuronal function and metabolic rate (Shimazu et al. 2013; Newman and Verdin 2017). Previous studies have illustrated that BHBA has neuroprotective effects by inhibiting neuroinflammation (Fu et al. 2015) and apoptosis (Cheng et al. 2013), and improving cognitive function (Wu et al. 2020). Recently, BHBA has been shown to ameliorate the deficiency of adult neurogenesis in the dentate gyrus and rescue hippocampal memory defects in a mouse model of Kabuki syndrome, a Mendelian intellectual disability syndrome caused by mutations in either of two genes (KMT2D and KDM6A) involved in chromatin accessibility (Benjamin et al. 2017). Increasing evidence suggests that BHBA could promote the expression of brain-derived neurotrophic factor (BDNF), a trophic factor associated with cognitive ability and neurogenesis (Marosi et al. 2016; Sleiman et al. 2016). These findings suggest that BHBA presents therapeutic potential for neurological disorders due to its neuroprotective properties.

Recently, we found that BHBA processed properties of excellent anti-inflammatory effects in heat stress-treated mice (Huang et al. 2022). Besides, the effects of heat stress on adult hippocampal neurogenesis and the underlying connections to cognitive dysfunction are unclear. The aim of this study was to determine the potential cellular and molecular mechanisms underlying neuroprotective effects of BHBA in heat stress-treated mice.

## Results

### BHBA improves spatial memory ability in the heat stress-treated mice

**Fig. 1:**
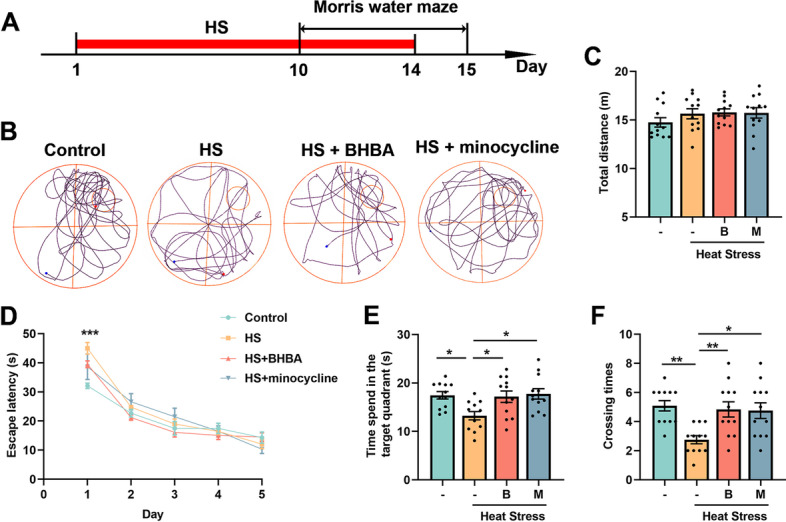
BHBA ameliorates spatial memory impairments in the heat stress-treated mice. **A** Experimental procedure of MWM. **B** Representative trajectories at day 6 of MWM. **C** Total swimming distance in the MWM. **D** Escape latency during the acquisition test (day 1–5). **E**–**F** Time spent in the target quadrant (**E**) and the number of platform crossings (**F**) in the prob trail on day 6. Both parameters decreased significantly in the heat-exposed mice and increased after treatment with BHBA or minocycline. For acquisition training (days 1 to 5) of the MWM, data was analyzed using a two-way ANOVA followed by a Tukey’s post-test to analyze the difference in escape latency between each group. Other data analyses were performed using one-way ANOVA with Tukey’s post-test. Data are represented as mean ± SEM. **C**-**F** *n* = 12. *, control *vs* heat stress. *p < 0.05, **p < 0.01, ***p < 0.001. B, BHBA; HS, heat stress; M, minocycline

Previous studies have demonstrated that heat stress could cause memory impairment in mice (Lee et al. 2015; Minho et al. 2017). Therefore, to investigate whether BHBA could alleviate heat stress-induced spatial learning and memory impairments, we conducted an Morris water maze (MWM) test to assess learning and memory ability in mice after heat exposure (Fig. 1A, B). In the spatial probe test performed on day 6, no significant change in total swimming distance was found, indicating no difference in locomotive activity among the groups (Fig. 1C). For the escape latency during the acquisition phase of MWM, the heat stress-vehicle treated mice exhibited learning deficits compared to mice in the control group on day 1. Heat stress did not cause a significant change in the mean of escape latency during the next four days of training in the MWM when compared to the control group. And on day 5 of the training, the escape latency for trained mice decreased in all groups, indicating the acquisition of spatial learning (Fig. 1D). However, according to the spatial probe test on the day 6, the swimming time within the target quadrant and hidden platform crossing times in the heat stress-vehicle treated group decreased significantly compared to those in the control group. On the contrary, the BHBA or minocycline-treated mice showed improved memory ability in MWM compared with heat stress-treated mice, manifested as increased time spent in the target quadrant and crossing times (Fig. 1E, F). These results suggest that spatial memory was protected by BHBA administration in the heat stress-treated mice.

### BHBA suppresses the reduction of synapse-associated proteins and dendritic spine density in the hippocampus of heat stress-exposed mice

**Fig. 2:**
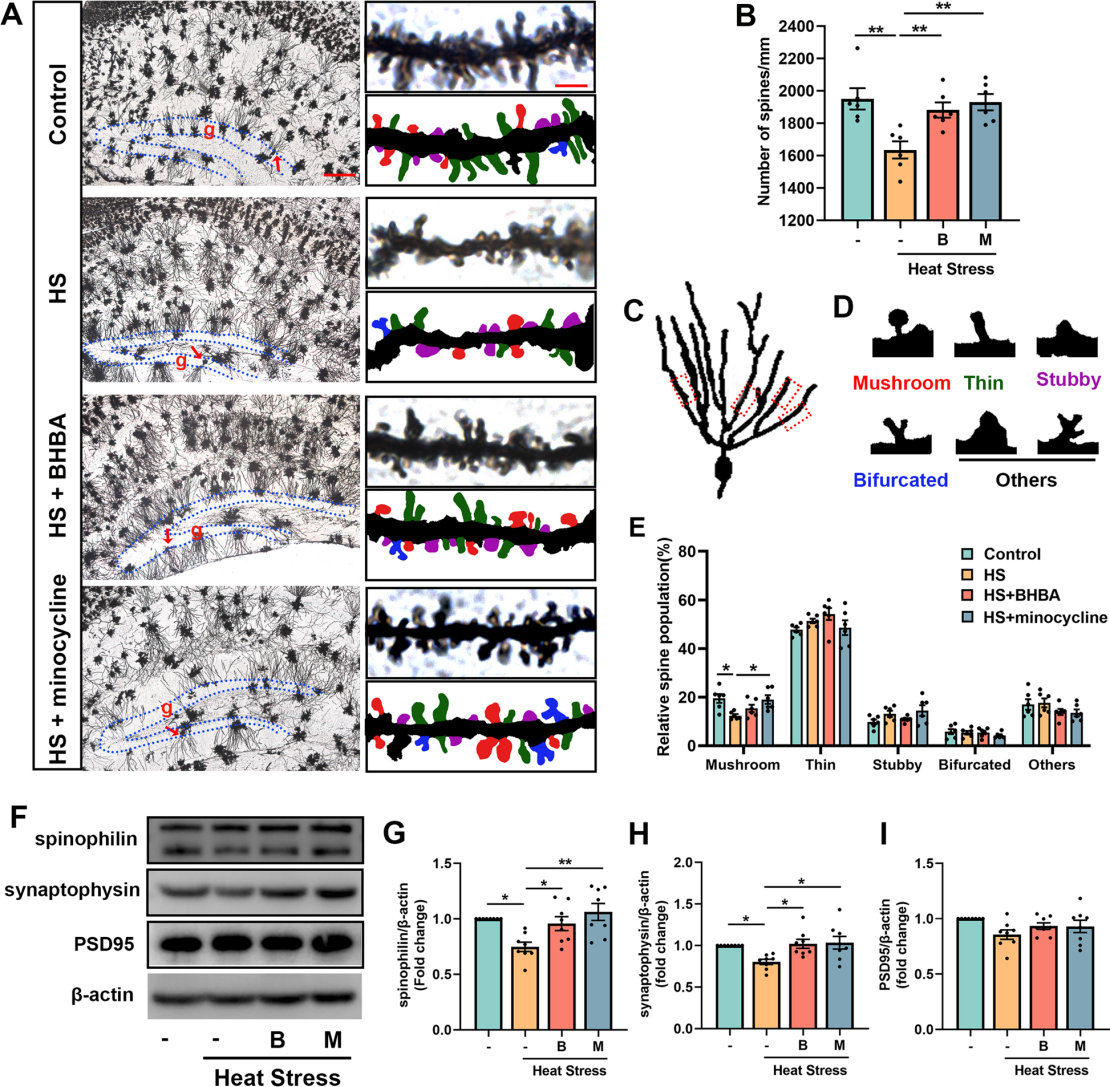
BHBA increases the density of dendritic spines and the expressions of synapse-associated proteins in the heat stress-treated mouse hippocampus. **A** Representative images of coronal sections subjected to Golgi staining from the hippocampus. Red arrows indicate granule cells in the hippocampal GCL (g, blue dashed box) of dentate gyrus. Dendritic reconstructions indicate the different types of spines according to their shape: mushroom (red), thin (green), stubby (purple), bifurcated (blue), and others (black). **B** Quantitative analysis of spine density of hippocampal granule cells. The spine density of granule cells was significantly lower in the heat stress-treated mice compared with that in the controls. BHBA or minocycline administration elevated spine density. **C** Representative morphology of granule cell of dentate gyrus with Golgi staining and the framed dendrite section for the analysis of spine density and morphology. **D** Representative photomicrographs of each type of spine. **E** Quantitative analysis of the classified spines in the hippocampal dentate gyrus. The proportion of mushroom spines decreased in the heat stress exposure group compared to that in the control group. And the proportion of other types of spines did not show a statistical difference among the four groups. **F** Immunoblotting images of spinophilin, synaptophysin, PSD95, and β-actin protein expressions in the hippocampus. **G-I** Quantitative densitometric analysis of spinophilin (**G**), synaptophysin (**H**), and PSD95 (**I**) in different groups. Mice treated with heat stress for 14 days showed significant decreases in the expressions of spinophilin and synaptophysin. After treatment with BHBA or minocycline, the expressions of spinophilin and synaptophysin showed remarkable increases in the heat stress-treated mice. Scale bar = 10 μm (left) or 2.5 μm (right) in **A**. Data were normalized to β-actin: expression of the target protein = intensity of target protein band/intensity of β-actin. The results are presented as the ratio of the experimental group to the control group with the value taken as 1. All data analyses were performed using one-way ANOVA with Tukey’s post-test. Data are represented as mean ± SEM. **B, E** *n* = 6 from 3 mice. **G-I** *n* = 8. *p < 0.05, **p < 0.01. B, BHBA; g, GCL; HS, heat stress; M, minocycline; PSD95, postsynaptic density protein 95

Incremental evidence suggests that synaptic dysfunction contributes to the deterioration of learning and memory performance (Arroyo-García et al. 2021). Dendritic spines are small, thin, specialized protrusions from neuronal dendrites and dendritic spine plasticity is an important mechanism underlying learning and memory (Frankfurt and Luine 2015). To determine the effects of heat stress on dendritic spines, we qualified the density and morphology of spines of granule cells in the hippocampal dentate gyrus using Golgi-Cox staining (Fig. 2A). For the analysis of spine density and morphology in this study, the dendrites in 15—30 μm lengths of the tertiary dendritic branches were used and two hippocampal granule cells per mouse from three mice were analyzed. As shown in Fig. 2B, we found a lower density of dendritic spines in the heat stress-treated mice relative to the control mice, which were reversed by BHBA or minocycline treatment. Meanwhile, the type of dendritic spines of tertiary segments from granule cells in the dentate gyrus was analyzed in the present study (Fig. 2C, D). The heat stress-treated mice showed a reduction in the ratio of mushroom spines. However, BHBA administration did not increase the ratio of mushroom spines in the heat stress-treated mice. No difference in the proportion of thin, stubby and bifurcated spines was found among the four groups (Fig. 2E).

Spinophilin is highly enriched in dendritic spines and appears to be required for the regulation of the properties of dendritic spines (Feng et al. 2000). The expression of spinophilin was statistically reduced in the hippocampus of mice treated with heat stress compared with that in the control group. Administration with BHBA or minocycline restored heat stress-induced decrease of spinophilin (Fig. 2G). Impaired synaptic plasticity is usually accompanied by decreased synaptic proteins, such as pre-synaptic marker synaptophysin and post-synaptic marker PSD95, which are important for synaptic function (Colnaghi et al. 2019). Compared with control mice, synaptophysin protein levels significantly decreased in the hippocampus of heat stress-treated mice. However, BHBA or minocycline treatment significantly attenuated the decrease of synaptophysin (Fig. 2H). No significant change was found among four groups in the expression of PSD95 (Fig. 2I). These findings suggest that BHBA may alleviate memory impairments by enhancing synaptic plasticity in heat stress-treated mice.

### BHBA enhances the proliferation of NSCs in the hippocampus of heat stress-treated mice

**Fig. 3:**
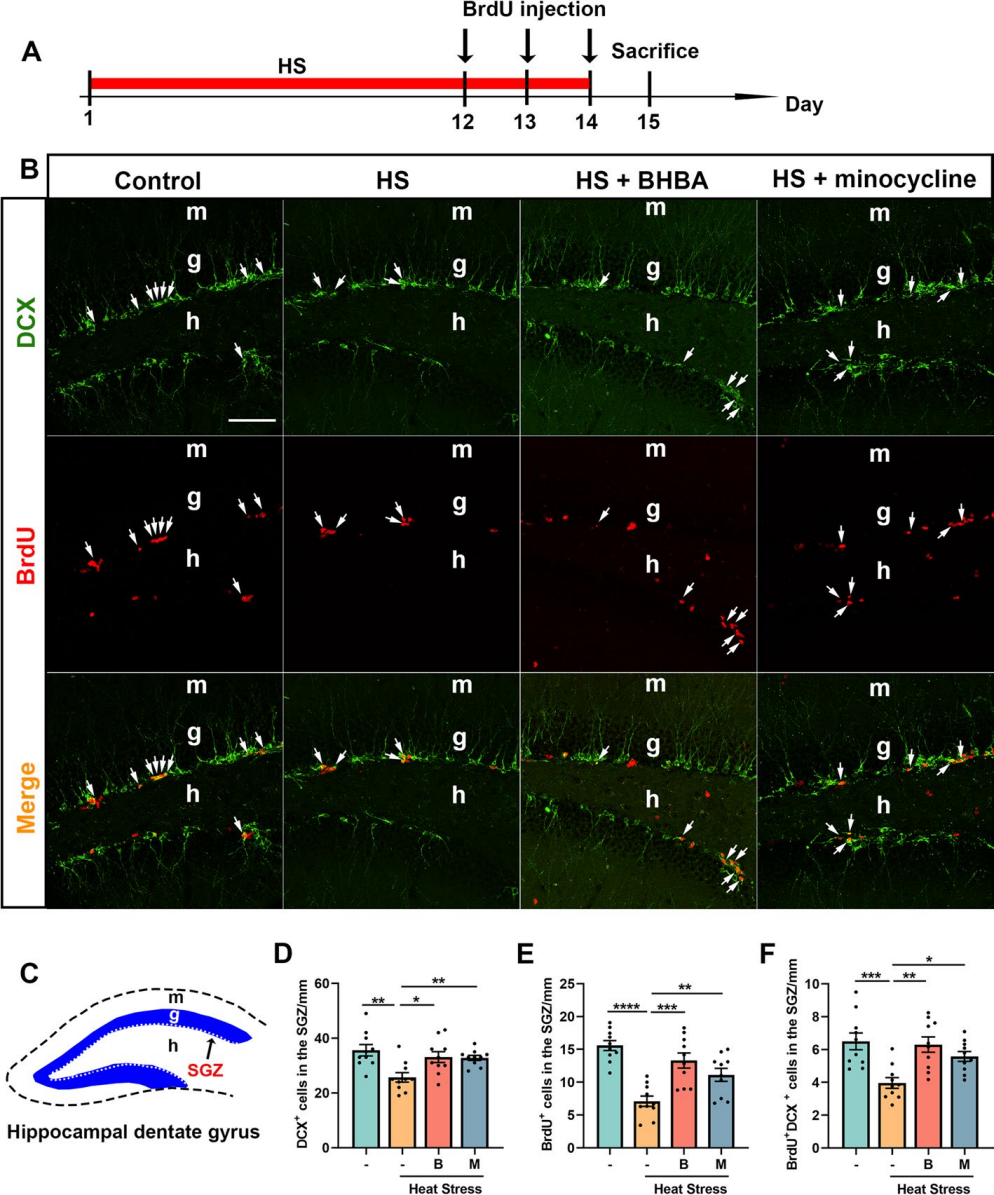
BHBA administration increases the proliferation of NSCs in the heat stress-treated hippocampal dentate gyrus. **A** Timeline of the experimental procedure. **B** Representative images are shown depicting the dentate gyrus sections immunostained with anti-BrdU (red) and anti-DCX antibodies (green). White arrows indicate BrdU^+^DCX^+^ double-labeled newly generated neurons in the dentate gyrus. **C** Schematic illustration of the dentate gyrus. The white dashed line at the border between the hilus and the GCL represents the SGZ. **D**-**F** Quantitative analysis of DCX^+^ cells (**D**), BrdU^+^ cells (**E**) and BrdU^+^DCX^+^ double-labeled cells (**F**) in the dentate gyrus. Compared with the control group, the density of DCX^+^, BrdU^+^ and BrdU^+^DCX^+^ cells decreased significantly in the heat stress-treated mice. Administration with BHBA or minocycline increased the densities of these cells. Scale bars = 50 μm. All data analyses were performed using one-way ANOVA with Tukey’s post-test. Data are represented as mean ± SEM. **D-F** *n* = 10 from 5 mice. *p < 0.05, **p < 0.01, ***p < 0.001, ****p < 0.0001. B, BHBA; g, granule cell layer; h, hilus; HS, heat stress; m, molecular layer; M, minocycline; SGZ, subgranular zone

NSCs in the hippocampus generate new neurons that integrate into existing hippocampal networks and modulate mood and memory (Austin et al. 2021). We, therefore, performed immunofluorescence to assay the level of proliferation of NSCs in the hippocampus. Mice received injections of BrdU twice per day during the last 3 days of heat exposure to label the proliferating cells which are in the S-phase of the cell cycle in the SGZ of the dentate gyrus (Fig. 3A-C). DCX is a marker of immature neurons in the adult hippocampus and the density of DCX^+^ cells in the GCL can be used as the index of hippocampal neurogenesis (Jin et al. 2002). Quantitative analysis showed that the density of DCX^+^ cells in the GCL was significantly decreased in the heat stress-treated mice compared to controls. Interestingly, administration with BHBA or minocycline increased the density of DCX^+^ cells in the GCL (Fig. 3D). We then assessed the total number of proliferative cells (BrdU^+^) as well as the newly generated neurons (BrdU^+^DCX^+^) along the entire SGZ in the dentate gyrus. We found that heat stress negatively influenced the density of BrdU^+^ cells and BrdU^+^DCX^+^ double-labeled cells compared with those in the control group and these dysfunctions were alleviated by BHBA or minocycline treatment (Fig. 3E, F). These results indicate that BHBA alleviates the impairments of hippocampal neurogenesis by promoting the proliferation of NSCs in heat stress-treated mice.

### BHBA amends the differentiation and survival of newly generated cells in the heat stress-injured hippocampus

**Fig. 4:**
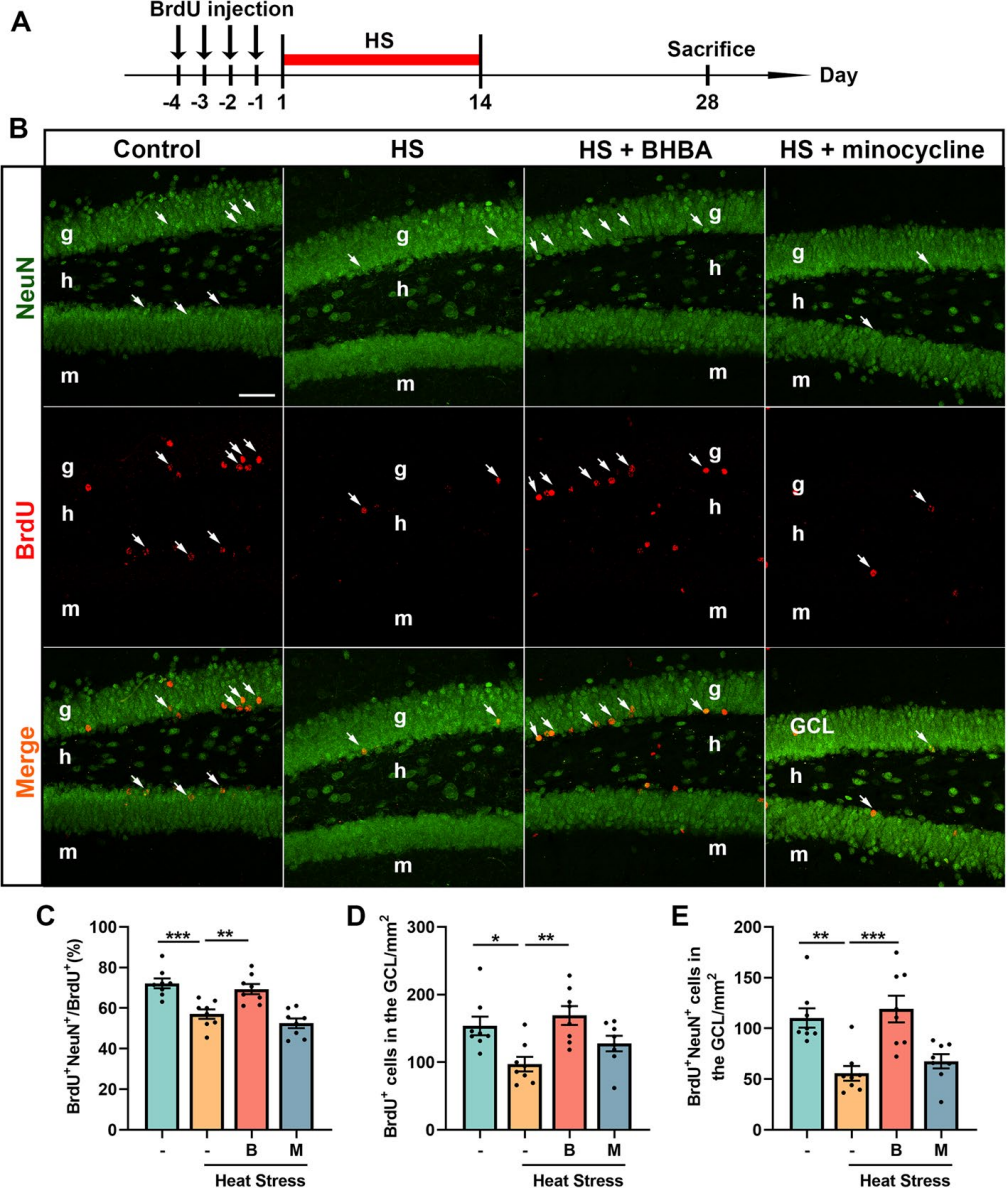
BHBA increases the neuronal differentiation and survival in the heat stress-treated mice. **A** Timeline of the experimental procedure. **B** Immunofluorescent photomicrographs of BrdU (red) and NeuN (green) in the dentate gyrus. White arrows indicate BrdU^+^NeuN^+^ newborn neurons in the GCL. **C** The ratio of BrdU^+^NeuN^+^ cells to total BrdU^+^ cells in the GCL. Heat stress treatment reduced the ratio of BrdU^+^NeuN^+^ cells to total BrdU^+^ cells, which was increased by BHBA treatment. **D**, **E** The number of BrdU^+^ cells (**D**) and BrdU^+^NeuN^+^ cells (**E**) in the hippocampal GCL. Heat stress-treated mice showed a reduced number of BrdU^+^ and BrdU^+^NeuN^+^ cells in the GCL. Administration with BHBA increased the number of BrdU^+^ and BrdU^+^NeuN^+^ cells in the heat stress-treated mice. Scale bars = 40 μm. All data analyses were performed using one-way ANOVA with Tukey’s post-test. Data are represented as mean ± SEM. **C-E** *n* = 8 from 4 mice. *p < 0.05, **p < 0.01, ***p < 0.001. B, BHBA; g, granule cell layer; h, hilus; HS, heat stress; m, molecular layer; M, minocycline

Following the proliferation of NSCs in the dentate gyrus, the newborn cells survive and differentiate into neurons or glial cells (Lazutkin et al. 2019). However, the effects of heat stress on the differentiation and survival of newly generated cells in the dentate gyrus of adult mice remains unclear. To investigate whether heat stress affects the differentiation of newly generated cells, mice were injected with BrdU for 4 days prior to heat stress to label the proliferating cells. The samples were collected on the 28^th^ day after the last BrdU injection (Fig. 4A). As shown in Fig. 4B, cells double-labeled with BrdU and NeuN (BrdU^+^NeuN^+^) represent the newly generated cells that differentiated into neurons. Compared with mice in the control group, a decreased percentage of BrdU^+^NeuN^+^ newborn neurons in entire newly generated cells was found in the heat stress-treated mice. BHBA treatment elevated the ratio of BrdU^+^NeuN^+^ newborn neurons in the heat stress-challenged mouse hippocampus (Fig. 4C). The survival of newly generated cells produced by adult neurogenesis is crucial for learning and memory functions (Cahill et al. 2017). In the present study, heat stress led to significant reductions in the numbers of total BrdU^+^ cells and BrdU^+^NeuN^+^ cells in the GCL compared with controls. In comparison with the heat stress-treated mice, the total number of BrdU^+^ cells and BrdU^+^NeuN^+^ cells was higher in the heat stress BHBA-treated group. However, minocycline treatment seemed to have no protective effects on the differentiation and survival of newly generated cells in the heat stress-treated mice (Fig. 4D, E). These findings demonstrate that BHBA ameliorated the differentiation of neurons and improved the survival of newborn cells in the heat stress-treated mice.

### BHBA promotes the migration of newborn neurons in the hippocampus injured by heat stress

**Fig. 5:**
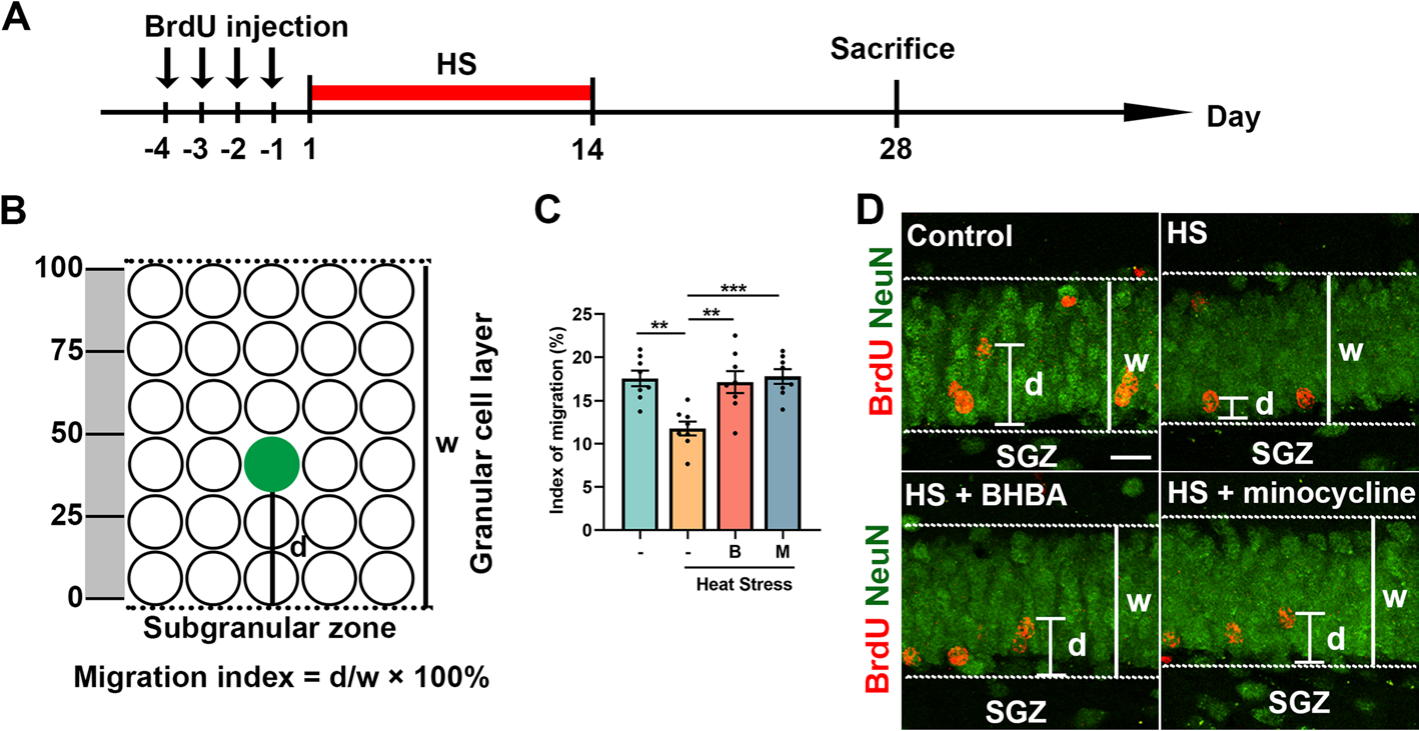
BHBA promotes the migration of newborn neurons in the hippocampus of heat stress-injured mice. **A** Protocol for testing the effect of heat stress on neuronal migration in the dentate gyrus. **B** Schematic representation of the calculation method of the index of neuronal migration through the GCL. The migration index was calculated using the formula, index of migration = d/w × 100%, where d represents the distance between the center of BrdU^+^NeuN^+^ cells and the SGZ, and w represents the width of GCL. **C** The graph displayed the index of migration. The index of migration was lower in mice with heat stress treatment, which was rescued by BHBA or minocycline treatment. **D** Representative confocal maximal projection images depicting the migration pattern of BrdU^+^NeuN^+^ cells through the GCL. Scale bar = 12.5 μm. All data analyses were performed using one-way ANOVA with Tukey’s post-test. Data are represented as mean ± SEM. **C** *n* = 8 from 4 mice. **p < 0.01, ***p < 0.001. B, BHBA; HS, heat stress; M, minocycline; SGZ, subgranular zone

Neuronal migration plays an essential role in hippocampus-dependent functions (Ming and Song 2005). To assess the effect of heat stress on the migration of newborn neurons in the dentate gyrus, BrdU was injected twice a day for 4 consecutive days before heat stress (Fig. 5A). The index of migration of newborn neurons was calculated by dividing the distance of BrdU^+^NeuN^+^ cells from the SGZ by the width of the GCL (Fig. 5B, D). The index of migration of BrdU^+^NeuN^+^ cells through the GCL with the treatment of heat stress was significantly lower than that in the control group. Surprisingly, BHBA or minocycline treatment increased the index of migration of BrdU^+^NeuN^+^ cells through the GCL compared with that in the heat stress-treated group (Fig. 5C). Taken together, these findings suggest that BHBA alleviated the inhibitory effect of heat stress on the migration of newborn neurons.

### BHBA restrains heat stress-induced apoptosis via inhibiting the eIF2α-ATF4-CHOP ER stress pathway

**Fig. 6:**
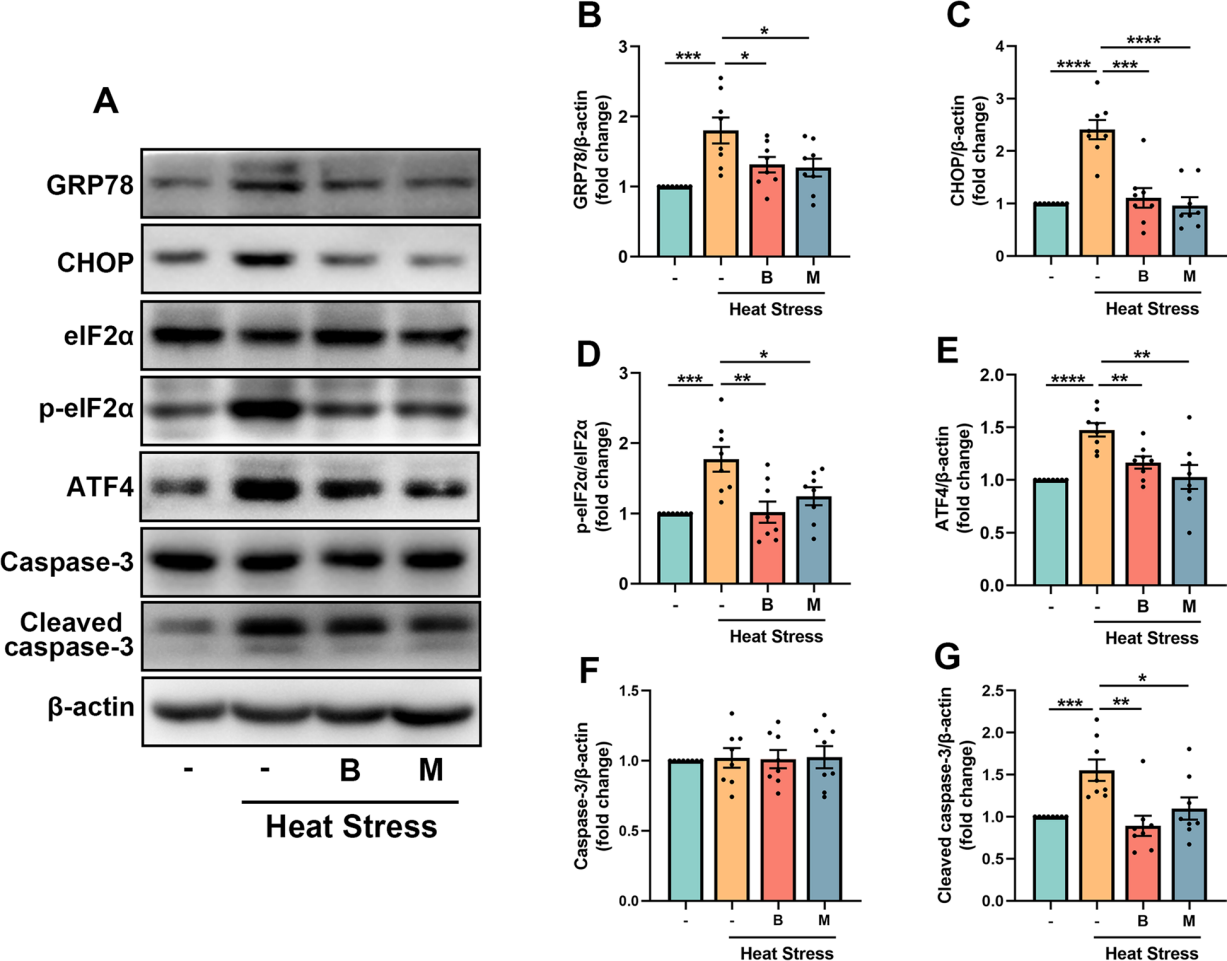
BHBA inhibits ER stress and cell apoptosis induced by heat stress. **A** Western blots of GRP78, CHOP, eIF2α, p-eIF2α, ATF4, caspase-3, cleaved caspase-3 and β-actin. **B**-**G** Relative expressions of GRP78 (**B**), CHOP (**C**), p-eIF2α/eIF2α (**D**), ATF4 (**E**), caspase-3 (**F**) and cleaved caspase-3 (**G**). Except for caspase-3, all above proteins increased significantly in the heat stress-treated mouse hippocampus and these elevated protein levels in the heat stress-treated mice were restored by BHBA or minocycline treatment. Data were normalized to β-actin: expression of the target protein = intensity of target protein band/intensity of β-actin. The results are presented as the ratio of the experimental group to the control group with the value taken as 1. All data analyses were performed using one-way ANOVA with Tukey’s post-test. Data are represented as mean ± SEM. **B-G** *n* = 8. *p < 0.05, **p < 0.01, ***p < 0.001, ****p < 0.0001. ATF4, activating transcription factor 4; B, BHBA; CHOP, C/EBP homologous protein; eIF2α, eukaryotic initiation factor 2α; GRP78, glucose-regulated protein 78; M, minocycline; p-eIF2α, phospho-eIF2α

In neurological diseases, the accumulation of misfolded proteins and concomitant induction of ER stress in neurons contributes to neuronal dysfunction (Hetz and Saxena 2017). In the present study, GRP78 and CHOP, two markers for ER stress, increased significantly in the heat stress-challenged mouse hippocampus, and treatment with BHBA or minocycline decreased their expressions following high-temperature challenge (Fig. 6B, C). To further explore the potential mechanisms, we assessed the levels of p-eIF2α/eIF2α and ATF4, which are correlated to the CHOP pathway (Sprenkle et al. 2017). We found that the levels of p-eIF2α/eIF2α and ATF4 increased significantly in the heat stress-treated group compared to those in the control group. Interestingly, the levels of p-eIF2α/eIF2α and ATF4 decreased significantly in the heat stress-BHBA or minocycline treatment group compared to those in the heat stress-treated group (Fig. 6D, E). ER stress is a well-established inducer for apoptosis initiation (Nishitoh 2012). Caspase-3, one of the key players in the caspase-mediated apoptotic signaling pathway, was examined in this study. No significant difference in the expression of caspase-3 was found among four groups in the hippocampus (Fig. 6F). However, in the heat stress-treated group, the expression of cleaved caspase-3 increased significantly compared to that in the controls. Importantly, a significant decrease in the expression of cleaved caspase-3 in the heat stress-treated mice was observed following administration with BHBA or minocycline (Fig. 6G). These results indicate that BHBA suppressed apoptosis induced by heat stress by regulating eIF2α-ATF4-CHOP signaling.

### BHBA increases the expression of BDNF by modulating Akt-CREB and MeCP2 pathways in the heat stress-treated mouse hippocampus

**Fig. 7:**
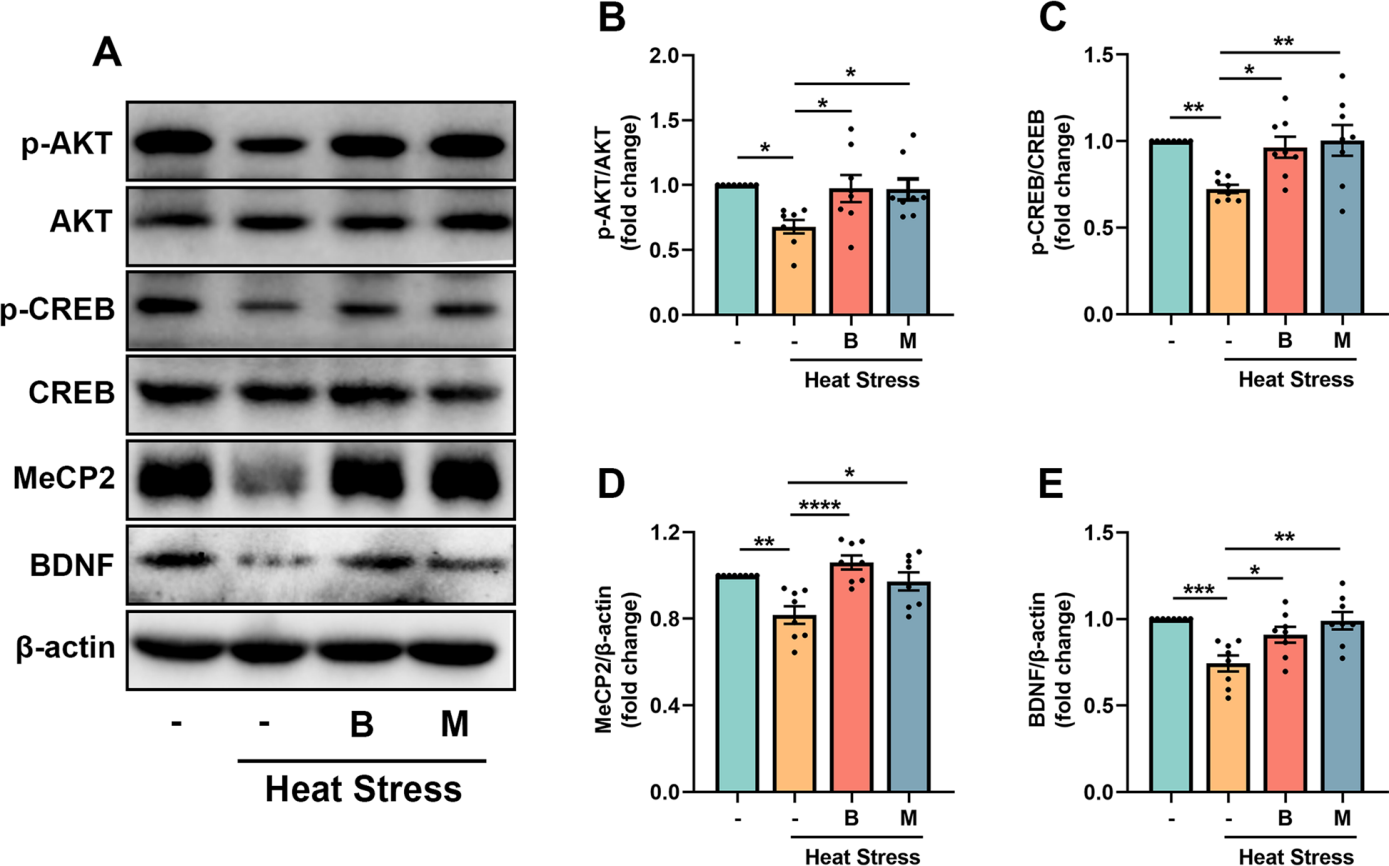
BHBA activates the Akt-CREB-BDNF and MeCP2 pathways in the heat stress-treated mice. **A** Representative western blot bands showing the relative protein expression of p-Akt, Akt, p-CREB, CREB, MeCP2, and BDNF. **B**-**E** Quantitative analysis showing the ratio of p-Akt/Akt (**B**), p-CREB/CREB (**C**), and the level of MeCP2 (**D**) and BDNF (**E**). Note that the phosphorylation of Akt and CREB and the expression of MeCP2 and BDNF were decreased in the heat stress-treated group, compared to the control group. However, BHBA treatment increased these protein levels in the heat stress-treated mice. Data were normalized to β-actin: expression of the target protein = intensity of target protein band/intensity of β-actin. The results are presented as the ratio of the experimental group to the control group with the value taken as 1. All data analyses were performed using one-way ANOVA with Tukey’s post-test. Data are represented as mean ± SEM. **B-E** *n* = 8. *p < 0.05, **p < 0.01, ***p < 0.001, ****p < 0.0001. Akt, protein kinase B; B, BHBA; BDNF, brain-derived neurotrophic factor; CREB, cAMP response element binding protein; M, minocycline; MeCP2, methyl-CpG binding protein 2

It is reported that phosphorylation of Akt could activate CREB, a transcription factor that regulates the transcription of BDNF (Esvald et al. 2020; Zarneshan et al. 2022). MeCP2 levels are also closely related to BDNF expressions (Chahrour et al. 2008). Western blot analysis illustrated significantly lower levels of phosphorylation of Akt and CREB in the heat stress-treated mice compared to controls. However, BHBA treatment increased the ratios of p-Akt/Akt and p-CREB/CREB (Fig. 7B, C). Heat stress treatment also significantly reduced the levels of MeCP2 in the hippocampus. Interestingly, BHBA or minocycline administration significantly attenuated the decreased MeCP2 levels (Fig. 7D). In addition, BHBA or minocycline significantly blocked the amelioration of decreased BDNF expression in the heat tress treated mouse hippocampus (Fig. 7E). These results suggest that BHBA may increase BDNF levels in heat stress-challenged mice through the regulation of Akt/CREB and MeCP2 signaling pathways.

## Discussion

**Fig. 8:**
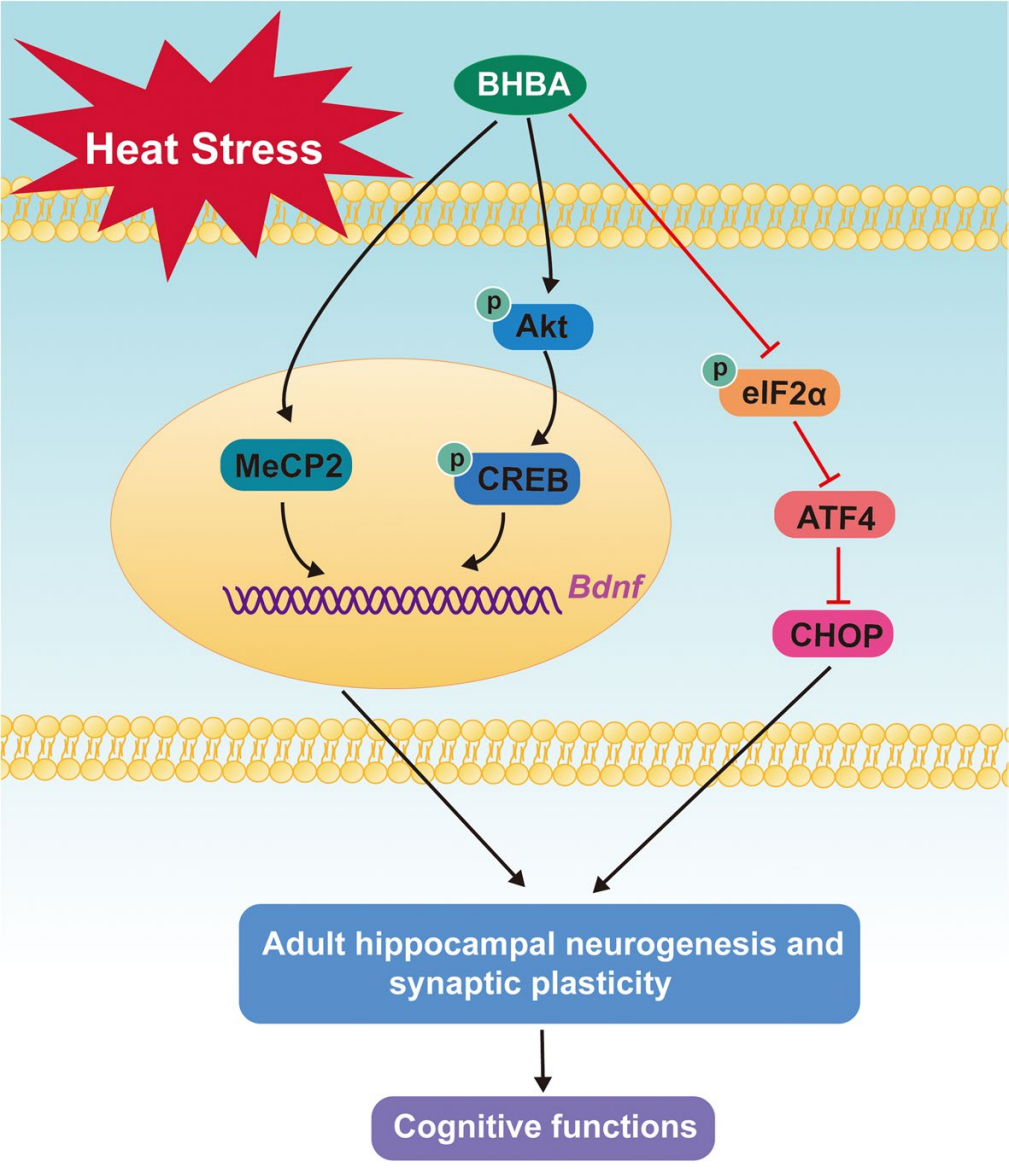
Schematic illustration of the possible protective mechanisms of BHBA administration in heat stress-induced cognitive dysfunctions in mice

Although several reports have described biochemical and neurophysiological changes occurring in the hippocampus following heat stress (Lee et al. 2015; Moon et al. 2017; Elvira et al. 2020; Huang et al. 2022), the impact of heat stress on adult hippocampal neurogenesis has been largely unaddressed. BHBA, a ketone body, has been shown to have neuroprotective roles in previous studies. Fu and his colleagues provided evidence that supports the effectiveness of BHBA in protecting dopaminergic neurons against inflammatory challenges (Fu et al. 2015). Also, BHBA improved the spatial learning of AD transgenic mice and attenuated the production and accumulation of Aβ (Wu et al. 2020). In the present study, we provided evidence of heat stress induced impairments in cognition and adult hippocampal neurogenesis, as well as the therapeutic role of BHBA. Here, we demonstrated that BHBA could increase BDNF levels by modulating MeCP2 and Akt-CREB pathways and inhibiting ER stress, which eventually reversed reduced synaptic plasticity and adult hippocampal neurogenesis (Fig. 8). This work extends previous results indicating the neuroprotective role of BHBA.

It is suggested that reduced synaptic plasticity might be involved in the impaired spatial memory ability (Wang et al. 2021). In this study, we found that heat stress resulted in spatial memory dysfunctions in mice, with less time spent in the target quarter and fewer platform crossings in the MWM, which is consistent with previous studies (Lee et al. 2015; Moon et al. 2017). A previous study reported that heat stress caused synaptic damage through decreasing synaptic markers (Erfani et al. 2019). According to Kim’s study, BHBA can preserve synaptic function in the hippocampus induced by mitochondrial respiratory complex dysfunction and this protective effect might be involved in its antioxidative activity (Kim et al. 2010). In this context, we investigated the effects of BHBA on the protein expression of synaptic plasticity markers in the hippocampus, such as PSD95, spinophilin, and synaptophysin. Heat stress decreased spinophilin and synaptophysin protein expressions, and the decreased expressions of spinophilin and synaptophysin were inhibited by BHBA treatment. Spinophilin is a scaffolding protein that is enriched in dendritic spines and plays an important role in regulating spine number (Areal et al. 2019). We found that heat stress significantly decreased the density of the dendritic spine in the hippocampal granule cells, which was paralleled by the changes in the decreased protein expression of spinophilin. The dendritic spine is the morphological component that neurons interact with each other through highly dynamic synaptic connections, and its changes in type and number processes are directly related to the formation and consolidation of memory (Seyer et al. 2020; Sun et al. 2020). Mushroom spines, which are proposed to form functionally stronger synapses and are responsible for memory storage (Bello-Medina et al. 2016), are decreased by heat stress in the present study. Our present findings revealed that BHBA treatment could block the decrease of synaptic plasticity induced by heat stress.

The relationship between hippocampus-dependent learning/memory function and adult hippocampal neurogenesis has been extensively investigated in recent years. Increased adult hippocampal neurogenesis facilitates spatial learning and memory (Shors et al. 2001). Conversely, decreased hippocampal neurogenesis is associated with impairments of memory (Winocur et al. 2006). Adult hippocampal neurogenesis, a multi-step process of the formation of new neurons in the brain throughout life in the SGZ, consists of proliferation of the NSCs, migration of neuroblasts, and differentiation into functional neurons (Ming and Song 2005). Results in the present studies demonstrated that heat stress affects all these stages, and administration with BHBA could alleviate the impairments of adult hippocampal neurogenesis stimulated by heat stress. In the current study, heat stress led to abnormal adult neurogenesis in the hippocampus, decreasing the DCX^+^ cells in the hippocampus, which is consistent with Lee’s study (Lee et al. 2015). Additionally, our results showed that heat stress decreased the number of BrdU^+^ cells in the SGZ, indicating a lower proliferation rate of NSCs. Interestingly, treatment with BHBA enhanced the proliferation of NSCs. Newborn neurons in the hippocampus of adult animals survive and develop into mature neurons, migrate and functionally integrate into the existing neural circuitry, which contributes to cognitive functions (Juliandi et al. 2015; Anacker and Hen 2017). Our study is the first to show that heat stress had adverse effects on the differentiation, survival, and migration of newly generated neurons in the hippocampus. However, the neuronal survival rate, the distance of migration, and differentiation were ameliorated by BHBA administration. BHBA is transported by monocarboxylate transporters to cross the blood–brain barrier and penetrates into the brain, and furthermore has been demonstrated to directly enter the hippocampus (Halestrap and Meredith 2004; Sleiman et al. 2016). As the natural end product of hepatic fatty acid beta-oxidation, BHBA has been shown to have histone deacetylase inhibitor activity (Shimazu et al. 2013). According to a previous study, exogenous BHBA treatment rescues the neurogenesis defect in a mouse model of Kabuki syndrome, and this effect may involve the promotion of chromatin opening (Benjamin et al. 2017).

BDNF is an important regulator of adult hippocampal neurogenesis and synaptic plasticity (Marosi et al. 2016; Nikoletopoulou et al. 2017), and exposure to heat stress decreases BDNF levels in the hippocampus in mice (Chauhan et al. 2021). BHBA could increase the expression of BDNF in mice or cultured cerebral cortical neurons (Marosi et al. 2016; Sleiman et al. 2016). As a serine/threonine kinase, Akt would be inactivated in response to heat stress (Li et al. 2019a, b). Of note, inactivation of Akt leads to the decreased phosphorylation of CREB, an essential transcriptional factor for BDNF, and ultimately decreases the BDNF expression in the hippocampus (Zarneshan et al. 2022). Interestingly, emerging evidence identified an important role of BHBA in the regulation of the phosphorylation of Akt (Carretta et al. 2020). Actually, in the present study, the reduction in the expression of BDNF induced by heat stress was accompanied by a decreased phosphorylation of Akt and CREB in the hippocampus. BHBA administration significantly attenuated the reduction in the expression of BDNF and the ratio of p-Akt/Akt and p-CREB/CREB in the heat stress-treated mice. MeCP2 is a transcriptional regular that is identified as the protein that binds to methylated CpG sites and plays a role as an activator on the *Bdnf* promoter (Nan et al. 1997; Chahrour et al. 2008). Consistent with this, Chahrour et al. demonstrated that the expression of BDNF is upregulated in MeCP2-overexpressed mice and downregulated in Mecp2-null mice (Chahrour et al. 2008). Similar to this report, we found that BHBA increased the expression of BDNF by elevating the levels of MeCP2 in the heat stress-treated mice. Based on these findings, it is suggested that the important mechanisms for improving adult hippocampal neurogenesis and synaptic plasticity of BHBA may be due to the activation of Akt-CREB-BDNF and MeCP2 pathways in the heat stress-treated mice.

Persistent ER stress has the potential to elicit inflammation and facilitate cell apoptosis (Sprenkle et al. 2017). Previous studies illustrated that heat stress caused ER stress and promoted the phosphorylation of eIF2α in the brain (Liu et al. 2012; Elvira et al. 2020), which is consistent with our present study. The global protein translation efficiency is decreased after the phosphorylation of eIF2α, but selectively enhances ATF4 transcription, which activates the downstream CHOP, a pro-apoptotic factor (Vattem and Wek 2004; Nishitoh 2012). Caspase-3 also can be activated in excessive ER stress conditions (Xu et al. 2018). In the present study, the protein levels of cleaved caspase-3 increased significantly in the hippocampus of heat-exposed mice and decreased after BHBA administration. Therefore, one could speculate that the increased survival rate of newly born cells under heat stress may be associated with BHBA-mediated anti-apoptosis effects. The eIF2α-CHOP is a classic pathway of ER stress-induced inflammation (Li et al. 2019a, b). The increased levels of p-eIF2α and ATF4 promote the activation of NF-κB, a prototypical proinflammatory signaling pathway, and ultimately upregulate the expression of IL-1β, TNF-α, and monocyte chemoattractant protein-1 (Zhang et al. 2015). Lee et al. illustrate that heat stress induces neuroinflammation and impairs adult neurogenesis in mice (Lee et al. 2015). Indeed, neuroinflammation is one of the widely recognized factors that cause cognitive dysfunction and impairment of adult hippocampal neurogenesis (Ryan and Nolan 2016). However, our previous study showed that BHBA has strong neuroprotective effects of anti-neuroinflammatory activities in heat stress-treated mice (Huang et al. 2022). In addition, a previous study has demonstrated that ER stress induces cognitive deficits and alterations in basal synaptic transmission, accompanied by decreased expression of synaptophysin and PSD95 in the hippocampus (Govindarajulu et al. 2020). However, as a potential neuroprotective drug, BHBA has been illustrated to exert a role in anti-ER stress and anti-inflammation (Fu et al. 2015; Tagawa et al. 2019). Unsurprisingly, in contrast to the heat stress-treated group, BHBA treatment down-regulated ER stress proteins in the current study. According to these findings, one of the possible mechanisms of promoting adult hippocampal neurogenesis and synaptic plasticity of BHBA might be involved in the anti-ER stress in heat stress-treated mice.

As a broad-spectrum tetracycline antibiotic and non-specific microglial inhibitor, minocycline has been demonstrated to suppress neuroinflammation induced by heat stress in mice (Huang et al. 2022). Meanwhile, minocycline could reverse the pathogenic phagocytic potential of neurotoxic M1 microglia, and reduces the negative phenotypes associated with reduced neurogenesis caused by exposure to chronic stress (Bassett et al. 2021). As the positive control group in this study, minocycline treatment increased spatial memory, proliferation of NSCs, migration of newborn neurons, and BDNF expression, and decreased ER stress-induced apoptosis in the heat stress-treated mice. However, minocycline administration did not increase the survival of newly generated neurons in the heat stress-exposed mice. Based on these findings, BHBA treatment appears to be more effective in inhibiting high temperature-induced brain injury.

The present study demonstrates that BHBA exerts its neuroprotective effects by inhibiting eIF2α-ATF4-CHOP ER stress and regulating Akt-CREB-BDNF and MeCP2 pathways in the heat stress-treated mice. These results reveal some new effects of heat stress on the hippocampus and the neuroprotective role of BHBA. In conclusion, from the aspects of synaptic plasticity and adult hippocampal neurogenesis, this study enriches the knowledge of the cellular and molecular mechanisms of impaired cognitive function induced by heat stress and proves the neuroprotective role of BHBA.

## Materials and methods

### Reagents and chemicals

BHBA (#54,965, Sigma-Aldrich, St. Louis, MO, USA) was originally dissolved in sterile saline to a concentration of 20 mg/mL. Minocycline (#S17015, Yuanye Bio-Technology Co., Shanghai, China) was dissolved in sterile saline to a concentration of 5 mg/mL. 5’-bromo-2-deoxyuridine (BrdU, #B5002, Sigma-Aldrich, St. Louis, MO, USA) was dissolved in sterile saline to a concentration of 5 mg/mL.

### Animals, heat exposure and drug treatment

Two-month-old ICR male mice were purchased from Xi’an Jiaotong University and housed in an adequate condition (24 ± 1 °C, 50% humidity) control with a 12 h/12 h light/dark cycle with free access to food and water for 1 week to adapt to the environment. Mice were divided into four groups randomly: control group (*n* = 32; sterile saline, 10 mL/kg, i.p.), heat stress group (*n* = 32; sterile saline, 10 mL/kg, i.p. + heat stress), heat stress BHBA-treated group (*n* = 32; BHBA, 10 mL/kg, i.p. + heat stress) and heat stress minocycline-treated group (*n* = 32; minocycline, 10 mL/kg, i.p. + heat stress). The dose of BHBA was chosen based on our previous study (Huang et al. 2022). The dose of minocycline was chosen based on Henry’s study (Henry et al. 2008). Minocycline is a broad-spectrum tetracycline antibiotic, used as an anti-inflammatory agent and microglia inhibitor, which could inhibit heat stress-induced cognitive deficits and neuroinflammation (Lee et al. 2015). Besides, minocycline can ameliorate the deficits of adult hippocampal neurogenesis (Liu et al. 2007). Heat stress minocycline-treated mice were used as the positive controls in the present study. Previous studies have illustrated that exposure to 43 °C for 15 min for 14 days could cause heat stress in mice (Lee et al. 2015; Huang et al. 2022). Therefore, in the present study, heat stress-treated mice received a daily intraperitoneal injection of different drugs and followed a 15 min heat exposure (43 ℃, 60% ± 10% humidity) in a chamber for 14 days 1 h after drug administration. To avoid the influence of diurnal cycling, heat exposure begins at 9 am each day.

### Morris water maze (MWM) test

The MWM test was performed to assess the spatial learning-memory of mice as described previously (Li et al. 2017). Briefly, a hidden platform was set under the surface (1.0 cm) of the water in the maze (120 cm in diameter × 35 cm in height) filled with water (22 ℃) mixed with black ink. Four different shapes were placed the on wall to help the mice locate the platform. Twelve mice from each group were trained for 5 consecutive days with 4 trials per day. Mice were released at four different locations in the maze, with their heads facing the wall, to search for the platform and stay on it for 20 s after reaching it. If the hidden platform is not found within 60 s, the mouse is guided to the platform. The time taken by every mouse to find and climb up the platform refers to escape latency. On the 6^th^ day, the hidden platform was removed from the maze and the mouse was released in the opposite quadrant to the target zone to explore the maze for 60 s. The number of crossing the platform and total time spent in the target zone were calculated by using a video camera and analyzed by ANY-maze software (Stoelting, Wood Dale, USA).

### BrdU labeling

BrdU labeling is the most common method for assessing cell proliferation as BrdU is incorporated into DNA during the synthesis phase of the cell cycle (Kee et al. 2002; Yan et al. 2018). To evaluate the effects of heat stress on the proliferation of NSCs, five mice from each group were injected intraperitoneally with 50 mg/kg BrdU twice per day during the last 3 days of the experimental schedule and the brains were harvested on the day after 24 h of the last BrdU injection. The early postmitotic maturation of newborn neurons occurs between day-7 and day-28 after the birth of adult-born granule cells (Song et al. 2012). To evaluate the impacts of heat stress on the survival, differentiation, and migration of the newly generated mature neurons, four mice from each group were injected intraperitoneally with BrdU twice a day for 4 consecutive days before heat exposure and the brains were harvested on the 28^th^ day after the last BrdU injection. Mice were deeply anesthetized using sodium pentobarbital (56 mg/kg) and transcardially perfused with saline followed by 4% paraformaldehyde in 0.1 M phosphate buffer (pH 7.4). The brains were post-fixed in 4% paraformaldehyde (PFA) at 4 ℃ until use.

### Immunofluorescence analysis

Coronal brain sections (thickness 50 μm) were obtained from the fixed brain immersed in PFA at least 3 d using a vibratome (VT 1000S, Leica, Germany) and rinsed with 0.1 M phosphate buffer saline for 3 times. For NeuN (1:500; MAB377, Millipore, Darmstadt, Germany) and DCX (1:500; sc-271390, Santa Cruz Biotechnology, Shanghai, China) with BrdU (1:500; MCA2060, Serotec, Düsseldorf, Germany) double-immunostaining, the sections were pretreated with 2 M HCl for 30 min at 37 ℃ and subsequently neutralized by 0.1 M borate buffer (pH 8.5), followed by rinsing with 0.1 M phosphate buffer saline for 3 times. The sections were incubated with primary antibodies which were diluted in a blocking solution at 4 °C overnight. Then sections were incubated with secondary antibodies as follows: Alexa Fluor 568 donkey anti-mouse IgG (1:500; A10037, Invitrogen), Alexa Fluor 647 donkey anti-rat IgG (1:500; A48272, Invitrogen), and Fluor 568 donkey anti-goat IgG (1:500; A11057, Invitrogen). Images were acquired using a laser-scanning confocal microscope (OLYMPUS FV3000, Japan). Stereological counts of the total number of positive cells were performed by an investigator blind to treatment by using ImageJ software (https://imagej.nih.gov/ij/index.html).

### Golgi-Cox staining

Golgi-Cox staining was performed to visualize the dendritic spines of hippocampal neurons according to the manufacturer’s protocol (FD NeuroTechnologies, USA). Briefly, three mice from each group mice were deeply anesthetized (sodium pentobarbital, 56 mg/kg, i.p.) and perfused with saline, then the freshly dissected brains were immersed in a mixture containing potassium dichromate and chromate at room temperature for 2 weeks and then transferred into Solution C, where they rested at room temperature for 72 h in a dark area. Coronal brain Sects. (100 μm thickness) were obtained using a vibratome. Sections were further transferred to Solution D and Solution E according to the manufacturer’s instructions. The brain sections were subsequently sensed in distilled water, dehydrated with sequential ethanol rising concentration baths (70% for 10 min, 90% for 10 min, 95% twice for 10 min, and 100% for 10 min), followed by a clarify in xylene solution (10 min). Finally, all sections were mounted with a resinous microscope medium. Images were acquired by using a Leica DM6 B microscope (Leica, Germany). Dendritic spines were classified into five different types: thin, mushroom, stubby, bifurcated and others (Tendilla-Beltrán et al. 2019). For the analysis of spine density and morphology, the dendrites in 15—30 μm lengths of the tertiary dendritic branches were used and two hippocampal granule cells in the granule cell layer (GCL) per mouse from three mice were analyzed in this study using ImageJ software by an investigator blind to treatment.

### Western blot analysis

Western blot was performed as described previously (Huang et al. 2022). Briefly, the hippocampus of mice was isolated on ice and homogenized in a RIPA buffer (Solarbio, Beijing, China) containing 1 mM PMSF (Solarbio, Beijing, China) and PhosSTOP EASYpack (Solarbio, Beijing, China), and then incubated on ice for 30 min, followed by centrifugation at 4 ℃ at 12,000 g for 25 min to obtain the total proteins. The protein concentration was measured using the BCA protein assay kit (Solarbio, Beijing, China). Total 20 μg protein was separated into 10% or 12% sodium dodecyl sulfate–polyacrylamide gel electrophoresis and transferred to polyvinylidene difluoride membranes (IPVH00010, Millipore, USA) for 2 h with 20% methanol in Tris–glycine buffer. The membranes were incubated with the primary antibodies at 4 ℃ overnight after blotting in the 5% non-fat dry milk in TBS containing 0.1% Tween-20 (Solarbio, Beijing, China). The following primary antibodies were used: rabbit anti-spinophilin (1:1000; ab18561, Abcam, Cambridge, MA, USA), mouse anti-synaptophysin (1:1000; ab32127, Abcam, Cambridge, MA, USA), rabbit anti-postsynaptic density protein 95 (PSD95; 1:1000; #2507, Cell Signaling Technology, Danvers, MA, USA), rabbit anti-eukaryotic initiation factor 2α (eIF2α; 1:1000; ab115822, Abcam, Cambridge, MA, USA), rabbit anti-phospho-eIF2α (Ser51; p-eIF2α; 1:1000; ab32157, Abcam, Cambridge, MA, USA), rabbit anti-C/EBP homologous protein (CHOP; 1:1000; BM4962, BOATER, Wuhan, China), rabbit anti-glucose-regulated protein 78 (GRP78; 1:1000; A0241, ABclonal, Wuhan, China), rabbit anti-activating transcription factor 4 (ATF4; 1:1000; BM5179, BOSTER, Wuhan, China), rabbit anti-caspase-3 (1:1000; #9662, Cell Signaling Technology, Danvers, MA, USA), rabbit anti-cleaved caspase-3 (1:1000; #9661, Cell Signaling Technology, Danvers, MA, USA), rabbit anti- protein kinase B (Akt; 1:1000; A17909, ABclonal, Wuhan, China), rabbit anti-phospho-Akt (Ser473; p-Akt; 1:1000; T40067, Abmart, Shanghai, China), rabbit anti-cAMP response element binding protein (CREB; 1:1000; #9197, Cell Signaling Technology, Danvers, MA, USA), rabbit anti-phospho-CREB (Ser133; p-CREB; 1:1000; #9198, Cell Signaling Technology, Danvers, MA, USA), rabbit anti-methyl CpG binding protein 2 (MeCP2; 1:1000; #3456, Cell Signaling Technology, Danvers, MA, USA), mouse anti-BDNF (1:1000; ab205067, Abcam, Cambridge, MA, USA), mouse anti-β-actin (1:1000; #3700, Cell Signaling Technology, Danvers, MA, USA). Then the membrane was incubated by the secondary antibodies at room temperature for 2 h. The following secondary antibodies were used: horseradish peroxidase-conjugated goat anti-rabbit IgG antibody (1:2000; #7074, Cell Signaling Technology, Danvers, MA, USA) or horse anti-mouse IgG antibody (1:2000; #7076, Cell Signaling Technology, Danvers, MA, USA). The reactive bands were visualized by enhanced chemiluminescence (ECL) detection kit (GE Healthcare, Buckinghamshire, United Kingdom) using the Q9 Alliance (UVItec, United Kingdom). Densitometric analysis was performed using the Q9 Alliance software. Western blot quantification was based on analysis of 8 replicate samples. Data were normalized to β-actin: expression of the target protein = intensity of target protein band/intensity of β-actin. The results are presented as the ratio of the experimental group to the control group, with the value taken to be 1.

### Statistical analysis

All data were expressed as mean ± SEM. All data were calculated using GraphPad Prism v.8.0 software (GraphPad Software, USA). All data analyses were performed using one-way ANOVA with Tukey’s post-test except for escape latency of MWM. For acquisition training (days 1 to 5) of the MWM, data was analyzed using a two-way ANOVA followed by a Tukey’s post-test to analyze the difference in escape latency between each group. *p* < 0.05 was considered as a threshold for statistical significance.

## Data Availability

The data and materials that support the findings of this study are available from the corresponding author upon request.

## Abbreviations

Akt: Protein kinase B
ATF4: Activating transcription factor 4
BHBA: β-Hydroxybutyric acid
BDNF: Brain-derived neurotrophic factor
BrdU: 5’-Bromo-2-deoxyuridine
CHOP: C/EBP homologous protei
CREB: CAMP response element binding protein
DCX: Doublecortin
eIF2α: Eukaryotic initiation factor 2α
ER: Endoplasmic reticulum
GCL: Granule cell layer
GRP78: Glucose-regulated protein 78
MeCP2: Methyl-CpG binding protein 2
ML: Molecular layer
MWM: Morris water maze
NSCs: Neural stem cells
p-Akt: Phosphor-Akt
p-CREB: Phospho-CREB
p-eIF2α: Phospho-eIF2α
PSD95: Postsynaptic density protein 95
SGZ: Subgranular zone
